# Glucose Serum Concentrations and Cardiovascular Disease in Patients on the End Stage of Renal Disease without Diabetes Mellitus

**DOI:** 10.3390/jcdd2020066

**Published:** 2015-04-24

**Authors:** Vaia D. Raikou, Despina Kyriaki

**Affiliations:** 1 Department of Medicine—Propaedaetic, National and Kapodistrian University of Athens, School of Medicine, Athens, 11527, Greece; 2 Department of Nuclear Medicine, General Hospital “LAΪKO”, Αthens, 11527, Greece; E-Mail: dkyriaki@gmail.com

**Keywords:** glucose, HOMA-IR, cardiovascular disease, hemodiafiltration

## Abstract

*Background/Aim*: It is still controversial whether tighter glycemic control is associated with better clinical outcomes in patients with kidney failure. We examined the association between glucose serum concentrations and cardiovascular disease in patients on the end stage of renal disease without diabetes mellitus. *Methods*: We studied 76 patients on on-line hemodiafiltration. Cardiovascular disease was defined by the existence of coronary disease (CD). Arterial stiffness was measured as carotid-femoral pulse wave velocity (c-fPWV) and carotid augmentation index (AIx). The concentrations of beta2-microglobulin (β2M) and insulin were measured by radioimmunoassays and insulin resistance by HOMA-IR. We built a logistic-regression analysis to examine the role of glucose on cardiovascular disease after adjustment for the traditional and specific risk factors for dialysis patients. *Results*: Serum glucose was positively correlated with beta2M, insulin and HOMA-IR (*r* = 0.361, *p* = 0.002, *r* = 0.581, *p* = 0.001 and *r* = 0.753, *p* = 0.001 respectively). Logistic-regression analysis did not show significant impact of glucose concentrations on cardiovascular disease after adjustment for traditional and specific risk factors. *Conclusions*: The association between elevated glucose serum concentrations and represented by coronary syndrome cardiovascular disease in patients on the end stage of renal disease without diabetes mellitus was not found significant.

## 1. Introduction

Poor glycemic control is associated with the development of comorbidities such as coronary artery disease and myocardial infarction in the general population [1]. Hyperglycemia has been shown to play a significant role on the development of microangiopathy and endothelial dysfunction [2]. Moreover, glycemia is known to influence the electrolyte balance, the function of potassium and calcium channels and sympathetic activity, all associated to arrhythmogenesis and sudden cardiac death of patients with kidney failure [3]. However, it is not clear whether patients on the end stage of renal disease (ESRD) with or/and without diabetes mellitus benefit from strict glycemic control [4].

In this study, we examined the association between glucose serum concentrations and cardiovascular disease in patients on the end stage of renal disease without diabetes mellitus.

## 2. Material and Methods

### 2.1. Subjects

We studied 76 hemodialyzed patients (hemodialysis mean duration =7.3 ± 6.0 years), 47 men and 29 women on mean age 62.2 ± 15 years. The treatment modality which was applied was on-line- predilution hemodiafiltration (on-l HDF). We excluded the patients with multiple intradialytic hypotensive episodes, fibrillation and the patients with interdialytic weight gain of > 5% of total body weight. Also, those with significant infection or malignancy were excluded from our study. The enrolled patients did not have interdialytic peripheral edema, high BP, interdialytic orthostatic hypotension or other characteristics of an inaccurate dry body weight.

The hemodialysis treatment was performed three-times weekly with a dialysis time of 3.5–4 h per session, a filter of 1.5–2 m^2^ surface area and a blood flow of 350–400 mL/min. A bicarbonate-based ultrapure buffer dialysis solution was used with a dialysate flow rate of 500–600 mL/min, a calcium concentration of 1.50–1.75 mmol/L, a sodium concentration of 138–145 mmol/L and low molecular weight heparin as anticoagulant therapy. We used high-flux synthetic membrane, defined by an ultrafiltration coefficient > 20 mL/h [5]. Dialysis dose was defined by Kt/V for urea, which was calculated according to the formula of Daugirdas [6]. The patients, who had Kt/V for urea < 1.2, were excluded from the study.

Cardiovascular disease was represented by the presence of coronary disease (CD, *n* = 25, 32.9%), which might be the underlying disease for heart failure or peripheral arterial disease.

The coronary syndrome was documented by history of myocardial infarction, coronary artery angioplasty or bypass surgery, clinical signs of angina pectoris or electrocardiographic ischaemic findings.

Patients with interdialytic blood pressure ≥ 160/90 (*n* = 29, 38.2%) were considered hypertensive, as well as the patients who were receiving anti-hypertensive drugs, such as calcium channel blockers, beta-blockers or inhibitors of agiotensin II receptors.

The family history for cardiovascular events or hypertension was positive at a ratio of 64.5% of the studied patients.

In our data, the renal failure was caused by hypertensive nephrosclerosis at a ratio of 33% and by chronic glomerulonephritis at a ratio of 30%.

### 2.2. Blood Collection

Blood samples were drawn just before the start of the mean weekly dialysis session in a 12 h fasting state from the vascular access. At the end of the treatment, the blood pump speed was reduced to < 80 mL/min and blood samples was obtained at 2 min post-dialysis from the arterial dialysis tubing for the calculation of the adequacy of dialysis by kt/V for urea. The blood samples were centrifuged, and kept at a temperature of −80 °C.

In each subject, three sequences of samples were received for the serum glucose measurements, and their average was used for statistical analysis.

### 2.3. Laboratory Measurements

Albumin, the ratio of low density lipoproteins (LDL) to high density lipoproteins (HDL) (LDL/HDL) and serum glucose concentrations were measured by biochemical analysis. Hematocrit, hemoglobin and monocytes blood cells values were also measured.

The concentrations of beta2-microglobulin, insulin and intact-parathormone (i-PTH) were measured by radioimmunoassays (Immunotech by Beckman, Prague, Czech Republic, BioSource Europe SA, Nivelles, Belgium and CIS bio international, Gif- sur-Yvette, France respectively).

Insulin resistance was calculated using the homeostasis model assessment of insulin resistance (HOMA-IR) [7].

High sensitivity C-reactive protein (hsCRP) and oxidized LDL (ox-LDL) serum concentrations were measured using enzyme linked immunoabsorbed assays (ΕLISA, Immundiagnostik AG., Bensheim, Germany) according to manufacturer’s specifications.

Normalized protein catabolic rate for dry body mass (nPCR) was calculated from the urea generation rate [8]. Body mass index (BMI) was obtained from height and post-dialysis body weight.

### 2.4. Hemodynamic Measurements

Predialysis peripheral systolic and diastolic blood pressures (SBP and DBP, respectively) were calculated as the mean of 10 measurements during a treatment month using an automatic sphygmomanometer OMRON M4-I (Co Ltd Kyoto Japan). Mean peripheral pre-dialysis BP (MBP) was calculated as: MBP = DBP + 1/3 (SBP − DBP). Before the mid-week dialysis session, the patients were allowed to rest for at least 10 min prior to their haemodynamic measurements. Arterial stiffness was measured as carotid-femoral pulse wave velocity (c-fPWV) and carotid augmentation index (AIx) using the SphygmoCor system^®^ (AtCor Medical Pty.Ltd, Sydney, Australia) according to manufacturer’s specifications. In each subject two sequences of measurements were performed, and their mean was used for statistical analysis. Pulse pressure (PP) was derived. Also, a 12-lead electrocardiographic examination was used to estimate the ischaemic findings.

## 3. Approval and Consent

The study was approved by the ethics committee of the Hospitals “Laiko, University General Hospital of Athens” and Renal Unit of “Diagnostic and Therapeutic Center of Athens Hygeia SA”. Written informed consent was obtained from all subjects.

## 4. Data Analysis

Data were analyzed using SPSS 15.0 statistical package for Windows (SPSS Inc, Chicago, IL, USA) and expressed as mean ± standard deviation or as median value ± interquartile range for data that showed skewed distributions; differences between mean values were assessed by using paired-t test and Mann-Whitney U test and expressed as mean ± standard deviation or as mean rank. Correlations between variables were defined by Pearson and Spearman coefficient and p values less than 0.05 were considered significant. ROC curve analysis was used for the determination of glucose cut off point related to coronary disease. x^2^ analysis was used for the correlation between categorical variables.We performed a logistic-regression analysis to investigate serum glucose concentrations as possible independent factor for cardiovascular disease after adjustment for the traditional and specific cardiovascular risk factors for dialysis patients, such as age, hypertension, BMI, dyslipidemia, anemia, inflammation, oxidative status, mineral bone disease, dialysis adequacy defined by Kt/V for urea and family history for cardiovascular disease.

## 5. Results

**Figure 1:**
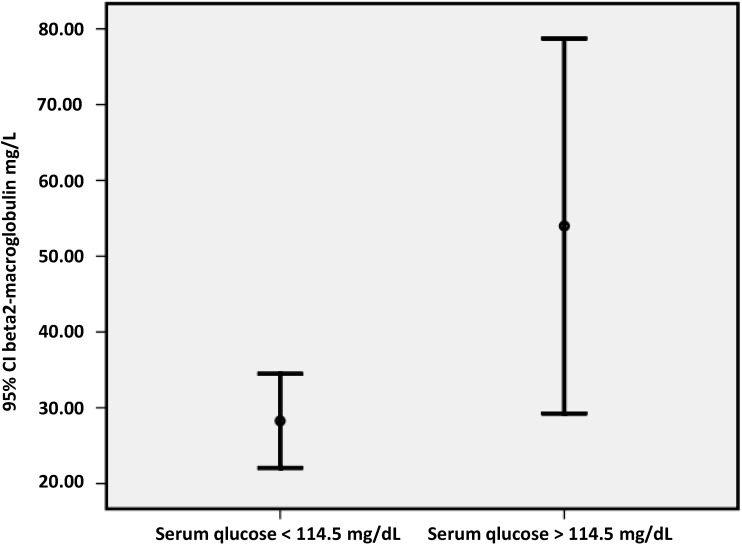
Beta2-microglobulin serum concentrations in patients on hemodiafiltration with glucose serum concentrations less and more than 114.5 mg/dL (*p* = 0.04).

We divided our patients in two groups according to found by ROC curve glucose cut off point related to coronary disease value equal to 114.5 mg/dL (greater, *n* = 18 or lower, *n* = 58 than 114.5 mg/dL). Characteristics and differences between two groups of patients are listed in Table 1. We observed that the patients with higher glucose concentrations (*n* = 18) had significantly increased insulin, HOMA-IR, hsCRP, PP values and beta2M serum concentrations than the patients with lower serum glucose values (*p* < 0.05) (Figure 1).

**Table 1:** Characteristics and differences between groups of patients according to glucose cut off point related to coronary disease (on-line hemodiafiltration, *n* = 76, *: *p* < 0.05).

Characteristic	Patients with Glucose Serum Concentrations Less than 114.5 mg/dL (*n* = 58, Mean ± SD or Mean Rank)	Patients with Glucose Serum Concentrations More than 114.5 mg/dL (*n* = 18, Mean ± SD or Mean Rank)
Age (years)	60.8 ± 14.5	67.6 ± 15.5
Dialysis duration (years)	37.8	36.4
BMI (Kg/m^2^)	24.2 ± 2.9	25.3 ± 3.1
Urine ( mL/day )	226.2 ± 150.6	166.6 ± 115.4
KTVurea	38.22	34.88
nPCR (g/Kg/day)	2.3 ± 0.4	2.5 ± 0.6
Glucose (mg/dL)	85.5 ± 13.7	132.7 ± 26.2 *
SBP (mmHg)	130.0 ± 20.7	138.3 ± 19.7
DBP (mmHg)	80.8 ± 10.2	84.3 ± 7.7
MBP (mmHg)	97.2 ± 12.9	102.3 ± 11.3
c-fPWV(m/s)	11.1 ± 1.8	12.08 ± 1.7
Augmentation index (AIx)	24.1 ± 2.3	24.4 ± 1.7
Pulse Pressure (PP, mmHg)	55.4 ± 17.7	66.7 ± 20.8 *
ABPI	1.12 ± 0.4	1.12 ± 0.5
Hemoglobulin	11.8 ± 1.3	12.01 ± 1.3
Monocytes (K/μL)	0.49 ± 0.2	0.50 ± 0.2
insulin (μU/mL)	32.7	54.7 *
ΗΟΜΑ-ΙR (mmol/L)	31.4	59.4 *
hsCRP (mg/L)	7.3 ± 6.03	10.6 ± 4.6 *
beta2-microglobulin (mg/L)	34.1	49.5 *
i-PTH (pg/mL)	38.2	34.6
Albumin (gr/dL)	37.9	36.09
LDL/HDL	2.3 ± 0.9	2.2 ± 0.7
ox-LDL (ng/mL)	35.2	45.8

### Correlations

**Figure 2:**
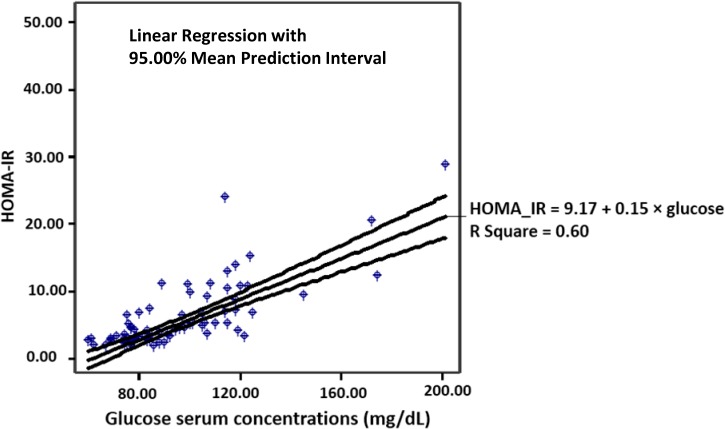
Correlation between glucose serum concentrations and insulin resistance defined by HOMA-IR (*n* = 76, *r* = 0.753, *p* = 0.001).

In total patients serum glucose was positively correlated with beta2M, insulin, HOMA-IR (Figure 2) and monocytes (*r* = 0.361, *p* = 0.002, *r* = 0.581, *p* = 0.001, *r* = 0.753, *p* = 0.001 and *r* = 0.272, *p* = 0.02, respectively). Also, HOMA-IR was significantly associated with both BMI and age (*r* = 0.303, *p* = 0.008 and *r* = 0.317, *p* = 0.005, respectively), although no significant association between glucose serum concentrations and neither BMI nor age could be found.

**Table 2:** Correlation between glucose serum concentrations and coronary disease in patients on hemodiafiltration after adjustment for risk factors.

Factors	B	S.E.	Sig.	Exp (B)
Glucose	−0.001	0.013	0.937	0.999
age	0.064	0.03	0.042	1.066
hypertension	1.042	0.75	0.169	2.836
BMI	−0.263	0.13	0.044	0.769
LDL/HDL	−1.288	0.55	0.020	0.276
Hb	0.605	0.32	0.060	1.832
hsCRP	0.092	0.06	0.164	1.097
oxLDL	0.011	0.005	0.025	1.011
i-PTH	0.004	0.002	0.025	1.004
Kt/Vurea	−5.816	2.9	0.044	0.003
Family history	0.099	0.824	0.905	1.104

The association of higher or lower serum glucose values with the presence of coronary disease was not found statistically significant by x^2^ analysis (*p* = NS). Also, the built logistic-regression analysis did not show significant impact of serum glucose concentrations on CD, after adjustment for the traditional and specific risk factors for these patients. However, the age, dyslipidemia, BMI, oxidative status defined by oxLDL, i-PTH and dialysis adequacy determined by kt/V for urea showed significant risk factors (Table 2).

## 6. Discussion

It has been reported that hyperglycemia induced generation of the inflammatory cytokines, oxidative stress and advanced glycation end products (AGEs). These toxins are profibrotic and directly involved in the pathogenesis of the vascular complications [9]. However, the link between intensive glycemic control and cardiovascular events is complex and still debated [10]. Some observational studies indicated the importance of good glycemic control, defined by serum glucose and hemoglobin A(1c), for cardiovascular disease in dialysis patients with diabetes mellitus (DM) [11].

Controversially, in several studies, no association between HbA1c, and neither patient survival nor cardiovascular disease could be shown in dialysis patients with DM [12]. Previous study of 1484 dialysis patients with or without DM showed that higher casual glucose and HbA1c levels were not associated with mortality in maintenance hemodialysis patients [13]. In agreement, we did not find a significant association between high glucose serum concentrations and coronary disease in our data, which included patients on hemodiafiltration without DM adjusting to traditional and specific for these patients’ factors.

In this study, we did not measure HbA1c concentrations, as it is known some inaccuracy of the HbA1c measurement in reflecting glucose levels in patients with chronic kidney disease. Studies suggest that glycated albumin is superior to HbA1c in estimating glucose control in dialysis patients [14].

Moreover, in the present study, we did not observe a significant difference for c-fPWV or AIx values in patients with higher glucose levels comparatively to the patients with lower glucose concentrations. c-fPWV and AIx values are used to assess arterial stiffness, which defines cardiovascular disease, and it has been reported that PWV varies during dialysis due to alterations in hydration status [15]. A previous study has reported that volume overload plays an important role in the development of arterial stiffness in HD patients [16]. Also, inflammation, mineral bone disease, hyperlipidemia and anemia have already been reported as specific cardiovascular risk factors for dialyzed patients, due partly to the relationship with arterial stiffness [17].

However, we observed significantly increased PP in patients with higher glucose than in patients with lower glucose levels. PP is influenced by both, arterial stiffness and hydration status, because blood pressure is mainly volume-dependent in these patients. This was related to PP extracellular volume, which might be extended in this group of patients comparatively to the patients with lower glucose concentrations, in whom the liquid balance may be achieved more effectively through dialysis treatment. This may explain the increased PP in the group of patients with higher glucose in comparison to the patients with lower glucose, rather than PP being associated with arterial stiffness.

On the other hand, in this study, the patients with higher glucose concentrations presented elevated insulin values, insulin resistance defined by HOMA-IR, significantly higher hsCRP and beta2M serum concentrations than the patients with lower glucose concentrations. Also, glucose was positively associated with beta2M, insulin, HOMA-IR and circulating monocytes.

It has been reported that hyperglycemia promotes inflammation by the excess generation of inflammatory cytokines and reactive free radicals, causing oxidative stress, which is associated with increased circulating inflammatory cells, elevated insulin resistance and the production of AGEs [2]. In the mean time, during hyperglycemia the generation of elevated AGEs modifies beta2M, which is accumulated in the circulation of dialysis patients [18]. AGE-modified beta2M (AGE-beta2M) may directly stimulate chemotaxis of monocytes and synthesis of cytokines from macrophages (interleukin-1β, TNF-α, and interleukin-6) [19]. Additionally, metabolic acidosis, which is a common condition particularly in end-stage renal disease patients [20] and which also promotes inflammation releasing cytokines [21], may be the connective pathophysiological mechanism between hyperglycemia, increased insulin resistance and increased accumulation of beta2Μ [22].

## 7. Conclusions

In this study, the association between elevated glucose serum concentrations and coronary syndrome cardiovascular disease in patients at the end stage of renal disease without diabetes mellitus was not found significant, despite that hyperglycemia was associated with extended extracellular volume and it promoted inflammatory procedure.
